# Short‐chain fatty acid delivery: assessing exogenous administration of the microbiome metabolite acetate in mice

**DOI:** 10.14814/phy2.14005

**Published:** 2019-02-27

**Authors:** Tyler B. Shubitowski, Brian G. Poll, Niranjana Natarajan, Jennifer L. Pluznick

**Affiliations:** ^1^ Department of Physiology Johns Hopkins School of Medicine Baltimore Maryland; ^2^ Department of Stem Cell and Regenerative Biology Harvard Stem Cell Institute Harvard University Cambridge Massachusetts

**Keywords:** Drinking water, i.p, injection, oral gavage, plasma acetate

## Abstract

Short‐chain fatty acids (SCFAs) are fermentation by‐products of gut microbes which have been linked to positive effects on host physiology; the most abundant SCFA is acetate. Exogenous administration of acetate alters host metabolism, immune function, and blood pressure, making it a biologic of interest. The effects of acetate have been attributed to activation of G‐protein–coupled receptors and other proteins (i.e., HDACs), often occurring at locations distant from the gut such as the pancreas or the kidney. However, due to technical difficulties and costs, studies have often delivered exogenous acetate without determining if systemic plasma acetate levels are altered. Thus, it is unclear to what extent each method of acetate delivery may alter systemic plasma acetate levels. In this study, we aimed to determine if acetate is elevated after exogenous administration by drinking water (DW), oral gavage (OG), or intraperitoneal (IP) injection, and if so, over what timecourse, to best inform future studies. Using a commercially available kit, we demonstrated that sodium acetate delivered over 21 days in DW does not elicit a measurable change in systemic acetate over baseline. However, when acetate is delivered by OG or IP injection, there are rapid, reproducible, and dose‐dependent changes in plasma acetate. These studies report, for the first time, the timecourse of changes in plasma acetate following acetate administration by three common methods, and thus inform the best practices for exogenous acetate delivery.

## Introduction

Recent studies have highlighted the important role of the gut microbiota in modulating host physiology. One mechanism utilized by microbiota to exert their influence on host physiology is the production of metabolites which influence signaling pathways in the host, often acting at sites in distant organs (Natarajan and Pluznick 2014, 2016). Notably, commensal microbes produce short‐chain fatty acid (SCFA) metabolites by the breakdown of dietary fiber (Hooper et al. 2002). The influence of SCFAs on host physiology has become an active area of investigation, and SCFAs have been shown to influence varied aspects of host physiology including immune responses, metabolism, and blood pressure control (Samuel et al. 2008; Maslowski et al. 2009; den Besten et al. 2013; Pluznick et al. 2013; Trompette et al. 2014; Mell et al. 2015; Yang et al. 2015; Raizada et al. 2017). SCFAs are one to six carbon chain volatile fatty acids, the most abundant being acetate, propionate, and butyrate (den Besten et al. 2013; Rios‐Covian et al. 2016; Panasevich et al. 2017). Once produced in the colon, SCFAs enter the systemic circulation via monocarboxylate transporters and passive diffusion (den Besten et al. 2013), where they can travel to distant sites and activate signaling pathways. While the mechanism of SCFA transport from the gut into the systemic circulation has been examined, it remains unclear how and at what rate SCFAs are cleared from the plasma. Although the precise levels and ratio of these three SCFAs can vary with diet, in all cases acetate is more abundant than either propionate or butyrate (Levrat et al. 1991; den Besten et al. 2013; Trompette et al. 2014).

To better understand the role of SCFAs in physiology, numerous studies have delivered SCFAs (including acetate) exogenously and then monitored physiological changes in the host (Egorin et al. 1999; Sakakibara et al. 2006; Yamashita et al. 2007; Ge et al. 2008; Kimura et al. 2011; Carvalho et al. 2012; Korecka and Arulampalam 2012; Lin et al. 2012; Frost et al. 2014; Ciarlo et al. 2016; Park et al. 2016; Jia et al. 2017; Mizuno et al. 2017; Li et al. 2018). Acetate is typically delivered by addition to drinking water (DW), by oral gavage (OG), or by intraperitoneal injection (IP). However, studies have only occasionally determined how systemic acetate levels are affected by exogenous acetate delivery, and usually only at one time point (Sakakibara et al. 2006; Yamashita et al. 2007; Ge et al. 2008; Frost et al. 2014; Lucas et al. 2018), largely due to the cost and blood volume necessary to perform this measurement. Typically, acetate is measured by electron ionization gas chromatography mass spectroscopy (EI‐GC MS) (Morrison et al. 2004; Samuel et al. 2008; Shepherd et al. 2014; Theriot et al. 2014; Tsukahara et al. 2014; Demehri et al. 2016; Mathewson et al. 2016; Perry et al. 2016; Tumanov et al. 2016; Zhao et al. 2016). Although EI‐GC MS is the current gold standard for acetate measurement, the use of this method to determine a “timecourse” of acetate changes in many model animals is restricted by the plasma volume required (~100 *μ*L). However, experimental design would be strengthened if the timecourse of changes in systemic acetate levels following delivery was better understood.

In this study, we took advantage of a commercially available Acetate Colorimetric Kit to detect in vivo changes in plasma acetate after exogenous sodium acetate delivery by either DW, OG, or IP. This kit, although not as sensitive as EI‐GC MS, requires only ~1 *μ*L of plasma and thus allowed us to determine a more detailed “timecourse” of acetate changes by performing repeated measurements in the same animal over time. Therefore, after examining the literature, we identified the most commonly used doses for each method of acetate delivery. Then, we experimentally determined how these doses and delivery methods modulate circulating plasma acetate, and over what timescale. These studies provide insight into the pharmacokinetics of acetate for the first time and serve to inform future experimental design.

## Methods

### Materials

Chemicals used include sodium formate (67253, Sigma), sodium acetate (S2889, Sigma), sodium propionate (P1880, Sigma), sodium butyrate (B5887, Sigma), valeric acid (240370, Sigma), sodium D‐lactate (71716, Sigma), and sodium L‐lactate (7022, Sigma).

### C57BL/6J mice

Procedures were in compliance with the NIH principles and guidelines for the Care and Use of Laboratory Animals, and policies and protocols were approved by the Johns Hopkins University Animal Care and Use Committee (ACUC). All mice used for this study were C57BL/6J originated from Jackson Laboratory (Bar Harbor, ME), and unless otherwise noted they were bred and housed for at least 6 months in the Johns Hopkins Miller Research Building (Baltimore, MD) on a 14/10‐hour light–dark cycle. A separate cohort of mice was used for each method of acetate administration. Unless otherwise specified, mice were treated between 11 and 13 weeks of age.

### Acetate delivery: Drinking water

DW was administered to mice on two different treatment schedules, both long (3 weeks) and short (24 h). During the long DW treatment, whole blood was first collected from C57BL/6J mice at 7 weeks of age as a baseline measurement. Whole blood was drawn from puncture of the submandibular vein with a lancet (Goldenrod) and collected in a lithium heparin tube (BD Microtainer^™^, 365965). Blood collections were immediately placed on wet ice until plasma separation. Mice were switched from an automatic water source to water bottle at this time. At 8 weeks of age, mice began the long treatment regimen with sodium acetate DW. Deionized water was mixed to a final concentration of 200 mmol/L sodium acetate, sterilized, and delivered to mice via ad libitum water bottle access (“acetate water”). Whole blood was then collected and processed following 7 and 21 days on acetate water (9 and 11 weeks of age), and weights were monitored during this time. After 21 days of acetate water, mice were switched back to regular water for 1 week. Whole blood was then collected again at Day 28 (12 weeks of age). Mice were sacrificed following this collection. All blood collections for this long DW treatment schedule were taken between 4 and 6 pm.

The collective absorbance mean of the baseline samples (Day 0) was set to one; all subsequent time points were averaged and normalized to baseline. A two‐way ANOVA with Sidak's multiple comparisons test was used to assay for changes in plasma acetate over the course of treatment and examine potential differences between the male and female mice.

In a separate cohort of mice, we collected blood to measure acetate via an EI‐GC/MS analysis. Mice used for the EI‐GC/MS analysis were ordered from Jackson Laboratories and were housed at Johns Hopkins for only 2 weeks before being given acetate (or control) water, as outlined below. For this experiment, 8‐week‐old mice were given either autoclaved deionized water or acetate water for 21 days. At the end of the experiment, a terminal blood sample was taken via retro‐orbital collection and then spun for plasma separation; all mice were sacrificed on the same day, and blood was collected between 10 am and 3 pm. SCFA analysis was then performed by the Michigan Regional Comprehensive Metabolomics Resource Core.

As plasma acetate did not appreciably change during the long‐term treatment, we also attempted a short‐term acetate DW treatment to try to maximize our ability to detect a change in plasma acetate. For this protocol, water was withdrawn for 24 h, and then, mice were given water supplemented with 200 mmol/L sodium acetate for 24 h. Whole blood collections were taken prior to deprivation and immediately before DW treatment, along with 0.25, 0.5, 0.75, 1, 2, 3, 5, 8, and 24 h after treatment began. Water deprivation began between 9 and 10 am and blood collections, the following day, began 24 h later (between 9 and 10 am), and concluded between 9 and 10 am on the third day.

For plasma collection for use in the Acetate Colorimetric Kit, whole blood drawn from tail tip excision was collected in lithium heparin capillary tubes (Sarstdt Microvette, 16.443.100). Blood collections were immediately placed on wet ice until plasma separation. This collection method yielded 6–10 *μ*L of whole blood per draw. The tail tip excision and draw enabled multiple collections over a short period of time (Ayala et al. 2010).

### Acetate delivery: Oral gavage

To determine if OG delivery of acetate modulates plasma acetate levels, we administrated sodium acetate by OG at two different doses (2 g/kg or 1 g/kg) between 8 and 10 am. Whole blood was drawn by tail tip excision at baseline and over time (0.25, 0.5, 1, 1.5, 2, 3, 5, 8, and 24 h following injection). Statistical analysis followed the method outlined for DW treatment.

### Acetate delivery: Intraperitoneal injection

SCFAs have also been delivered to mice via IP injection (Ge et al. 2008; Kimura et al. 2011; Frost et al. 2014; Ciarlo et al. 2016). To determine if IP injection of sodium acetate modulates systemic acetate levels, we administrated sodium acetate IP at two different doses (1 or 0.5 g/kg). IP administrations took place between 8 and 10 am unless otherwise noted. Urine samples were collected from mice following acetate administered via IP injection at the 1 g/kg dose. Mean arterial pressure (MAP) measurements were taken following acetate administered via IP injection at the 1 g/kg dose.

Whole blood was drawn by tail tip excision at baseline and over time (5, 15, 30, and 45 min along with 1, 2, and 3 h following injection). Plasma samples were prepared as described above. Statistical analysis followed the method outlined for the DW treatment.

For urine collection, mice were placed on Labsand (Braintree Scientific) a hydrophobic, nontoxic sand that allows mice to remain in standard housing, with ab libitum access to food and water. Mice were housed individually for 2.5 h and urine, when present, was collected at 15 min intervals.

In both cases, neither the baseline plasma nor baseline urine absorbance values were significantly different between the male and female mice of their respective cohort; therefore, the mean of these respective baseline values was set to one, and all subsequent time points were normalized to either the plasma or urine baseline. Statistical analysis was performed by one‐way ANOVA with multiple comparisons via Tukey's posttest.

### Plasma and urine sample preparation and storage

Whole blood was spun at 4°C for 10 min at 9300 *g*, and plasma samples were aliquoted and stored at −80°C until an assay was run. Special care was taken when collecting samples to minimize the extent to which whole blood was hemolyzed, as the change in color associated with hemolysis appeared to artificially increase the absorbance recorded in the Acetate Colorimetric Assay. All mouse plasma samples were visibly inspected to ensure that hemolysis was not present. Urine was collected and kept at 4°C until an assay was performed. Prior to use, urine was spun for 30 sec at 9300 *g* to pellet any Labsand that might have been present in the collection tube.

### Acetate colorimetric assay

The Acetate Colorimetric Assay Kit (MAK086, Millipore Sigma) was used to detect acetate abundance. In this proprietary coupled enzyme assay, acetate in the sample is converted into an intermediate in the presence of the Acetate Enzyme Mixture and Acetate Substrate Mix. This intermediate reduces the probe to a colored product with a strong absorbance at 450 nm; the colored product is proportional to the amount of acetate in the sample. We used this assay to measure the absorbance of the resulting colorimetric product using a FLUOstar Omega microplate reader (BMG labtech) at absorbance 450 nm following 40 min of incubation. The provided protocol for this kit was followed directly and states that it can be used to detect acetate in a variety of biological samples including serum, tissue, cells, and food. No additional sample purification steps were taken.

All reactions were 50 *μ*L in total volume and run in duplicate in a 96‐well plate. 40 *μ*L of this reaction mixture contained either 2 *μ*L of Acetate Enzyme Mixture (“test condition”) or 2 *μ*L of Acetate Assay Buffer (“blank condition”). The resulting test condition reads were corrected to remove background signal by subtracting the blank condition. Each reaction contained 1 *μ*L of mouse plasma diluted in 9 *μ*L of Acetate Assay Buffer. This 10 *μ*L of dilute mouse plasma was added to the corresponding 40 *μ*L of reaction mixture to reach the final reaction volume. All data are plotted as mean ± standard error.

Specificity for acetate was demonstrated by adding known amounts of acetate, other SCFAs, or lactate. Individual SCFAs or lactate were dissolved in Acetate Assay Buffer at varying concentrations up to 1 mmol/L and lactate up to 10 mmol/L. All tests for enzyme specificity were performed in the presence of 1 *μ*L of mouse plasma from 7 week‐old, cohabitated, untreated, C57BL/6J mice. Statistical significance was determined by a one‐way ANOVA of all doses within each compound.

### Blood pressure telemetry

Male mice, 11 weeks of age, were implanted with radio telemetry devices (PA‐C10, Data Science International) in order to measure MAP. The method of implantation follows protocols previously used in our laboratory (Natarajan et al. 2016). Mice were allowed 7 days for recovery. MAP was recorded for 10 min until mice were injected IP with 1 g/kg sodium acetate or a matched volume of saline. Averaged minute‐by‐minute recordings of MAP then proceeded until blood pressure returned to baseline, and saline and acetate were compared at each timepoint with a *t*‐test using a Bonferroni correction for multiple comparisons.

## Results

### Acetate‐specific colorimetric assay

**Figure 1:**
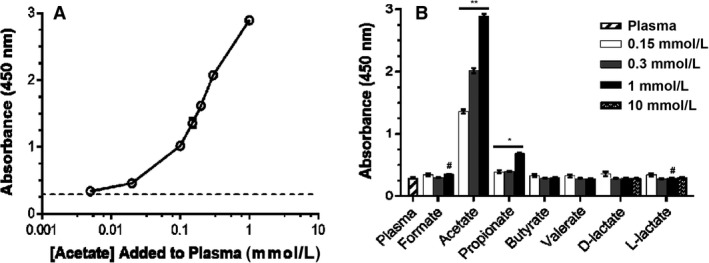
Acetate Colorimetric Kit demonstrates specificity for acetate. Adding exogenous acetate directly to plasma results in an increase in absorbance (A). This robust increase in absorbance is only seen when acetate is added to plasma (B: “plasma” indicates plasma alone, all other bars are plasma + indicated compound). Data shown as mean ± SEM of triplicate wells. Acetate: all pairwise comparisons are significant, *P *<* *0.001 (indicated by **); Propionate: 1 mmol/L dose significantly different from all other doses (*P *<* *0.001), 0 mmol/L significantly different from 0.15 and 0.3 mmol/L (*P *=* *0.003) (indicated by *). Formate: *P *<* *0.04 for 0 mmol/L versus 1 mmol/L (indicated by #). L‐lactate: significant *decrease* in absorbance with increasing dose (*P *<* *0.04 for 0.15 mmol/L versus 0.3 mmol/L; indicated by #).

The Acetate Colorimetric Assay Kit (MAK086, Sigma) was validated using concentrations of sodium acetate from 5 *μ*mol/L to 1 mmol/L (Fig. 1A). We further validated the specificity of this kit by demonstrating a concentration‐dependent increase in absorbance at 450 nm when a known concentration of acetate was added to a mouse plasma sample (Fig. 1B). However, this robust increase in absorbance was absent for other SCFAs (formate, butyrate, or valeric acid) or either D or L lactate. There was, however, a small but significant increase in absorbance with increasing doses of propionate (see figure legend), indicating that the kit may also react with propionate at higher concentrations.

Although a standard curve can be used to convert absorbance values to *μ*mol/L values, when we fit absorbance values for baseline mouse plasma to this standard curve, the calculated concentration of acetate ranged from −7 to 14 *μ*mol/L. This is less than other reported baseline concentrations of acetate in mice, which are generally reported to be ~0.1 mmol/L (Perry et al. 2016); furthermore, ~25% of our baseline samples yielded negative concentrations of acetate when fit to the standard curve. Therefore, because the goal of our study was not to quantify baseline levels, but to determine the timecourse of changes in systemic acetate over baseline after exogenous delivery, all data using the Acetate Colorimetric Assay are presented as fold change relative to baseline. This method of normalization allows for visualization of significant elevations in systemic plasma acetate over baseline. Of note, we did not observe any differences in baseline values between males and females.

### Drinking water treatment

**Figure 2:**
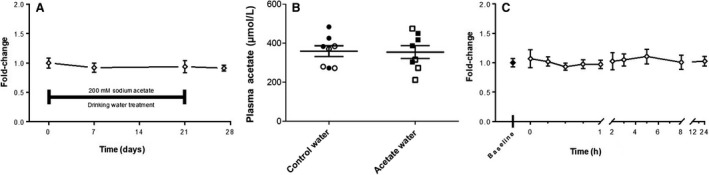
Plasma acetate does not change following sodium acetate DW treatment. Plasma was collected from nine mice (*n *=* *3 females, six males) at four timepoints over 28 days; mice were treated with 200 mmol/L sodium acetate DW from Day 0–21 and plasma acetate was measured using the Acetate Colorimetric Kit (A). In a separate experiment, plasma was collected from eight mice (*n *=* *4 females, open symbols; *n *=* *4 males, closed symbols) after 21 days of treatment with either control water or acetate water. Plasma acetate was then measured by EI‐GC/MS (B). Finally, plasma was collected from eight mice (*n *=* *4 females and four males) prior to and immediately following a 24 h water deprivation, along with at nine timepoints over 24 h while treated with 200 mmol/L sodium acetate DW (C); acetate was measured using the Acetate Colorimetric Kit. For C, all timepoints are *n *=* *8 with the exception of 5 h (*n *=* *7) and 8 h (*n *=* *6), due to technical difficulties. There were no significant differences present at any of the time points, between males or females, in any of the treatment conditions. DW, drinking water.

Administration of sodium acetate via DW is a noninvasive, passive method to deliver acetate to mice. To determine if doses and timecourses used previously in the literature (Park et al. 2016; Mizuno et al. 2017) would elevate systemic acetate levels over baseline, nine mice were given free access to water containing 200 mmol/L sodium acetate and plasma samples were collected at 0, 7, and 21 days of treatment as well as 7 days posttreatment. There was no significant change in plasma acetate for either sex from baseline (Day 0), through treatment (Days 7, 21), or when compared to the posttreatment collection (Day 28) (Fig. 2A). No weight loss was seen over this timecourse, and in a parallel study, we found that mice drank similar volumes of acetate water versus control water. To rule out a low sensitivity of the Acetate Colorimetric Assay as the reason for the lack of change in plasma acetate, in a separate cohort of mice, we collected blood samples after 21 days on either control water or acetate water and measured plasma acetate via EI‐GC MS (Fig. 2B). Again, we saw no change in plasma acetate with acetate water treatment nor did we observe changes in plasma propionate or butyrate. However, we reasoned it was still possible that there may be a transient elevation in systemic acetate that we may miss with weekly measurements.

To address this, we undertook an experiment in which mice were deprived of DW for 24 h and then given free access to water containing 200 mmol/L sodium acetate, to encourage the mice to consume water in a specific time window. A transient weight loss was seen during the water deprivation treatment; however, we found that the weight recovered to a similar extent when mice were given acetate water or (as a separate control) control water. Whole blood was collected and analyzed prior to deprivation, immediately prior to acetate treatment, and at nine timepoints following acetate treatment. There was no significant change in plasma acetate for either sex between hydrated and water deprived states or between these baseline measurements and any of the other timepoints (Fig. 2C).

### Oral gavage

**Figure 3:**
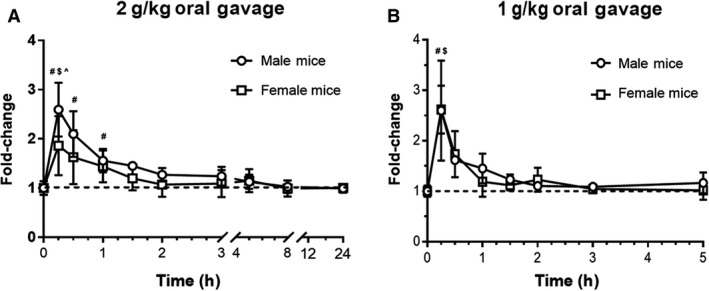
Plasma acetate increases following acetate oral gavage. Sodium acetate was delivered by gavage at 2 g/kg (A; *n *=* *4 male, *n *=* *4 female) and 1 g/kg (B; *n *=* *4 male, *n *=* *4 female) following a baseline collection. This method of delivery significantly elevated plasma acetate at both doses and in both sexes. Baseline is indicated by a dotted line; #denotes significant change from baseline in male acetate value, $denotes significant change from baseline in female acetate value, ^denotes significant difference between males and females.

SCFAs have also been delivered to mice via OG at a variety of doses (Egorin et al. 1999; Carvalho et al. 2012; Korecka and Arulampalam 2012; Jia et al. 2017; Li et al. 2018). When we administered 2 g/kg acetate to mice, plasma acetate levels elevated significantly, peaking at 15 min after administration in both male and female mice (Fig. 3A). Systemic plasma acetate values remained significantly elevated at 30 and 60 min after administration in males. When comparing plasma acetate level differences between the two sexes, a significant difference was present at 15 min. Subsequent blood draws after 60 min were not significantly elevated above baseline for either sex. By 8 h, plasma acetate levels were at or very near baseline in both sexes.

A similar result was observed with a 1 g/kg dose; however, the return of plasma acetate toward baseline was more rapid (Fig. 3B). Plasma acetate levels were significantly elevated above baseline in both males and females at 15 min post‐injection. By 30 min post‐injection, neither sex had acetate levels significantly above baseline. By 3 h, plasma acetate levels were at or very near baseline in both sexes.

### Intraperitoneal injection

**Figure 4:**
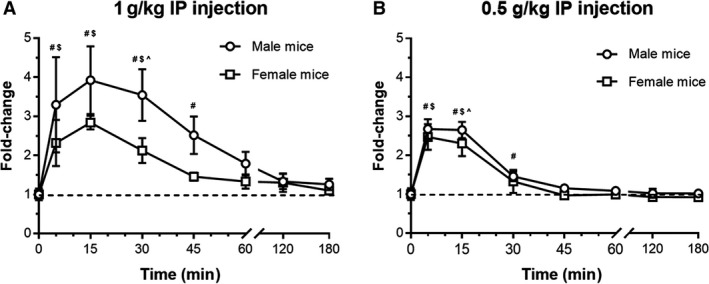
Plasma acetate increases following acetate IP injection. Sodium acetate was delivered IP at 1 g/kg (A; *n *=* *5 male, *n *=* *4 female) and 0.5 g/kg (B; *n *=* *4 male, *n *=* *4 female) following a baseline collection. This method of delivery significantly elevated plasma acetate at both doses and in both sexes. Baseline is indicated by a dotted line; #denotes significant change from baseline in male acetate value, $denotes significant change from baseline in female acetate value, ^denotes significant difference between males and females.

Another mode of delivery commonly used to modulate systemic acetate in mice is IP injection. When acetate was administered by IP injection at 1 g/kg (a dose used in the literature, [Ciarlo et al. 2016; Kimura et al. 2011]), plasma acetate levels elevated significantly, peaking at 15 min after injection in both male and female mice (Fig. 4A). Plasma acetate values remained elevated at 5, 15, and 30 min after injection in both males and females. Additionally, the male mice had significantly elevated plasma acetate levels 45 min after injection, and there was a statistically significant difference in the plasma acetate levels between the two sexes at 30 min. Subsequent blood draws after 45 min were not significantly elevated above baseline for either sex. By 3 h, plasma acetate levels were at or very near baseline in both sexes.

A similar result was observed with the 0.5 g/kg dose (also cited in the literature, [Frost et al. 2014; Ge et al. 2008]); however, the return of plasma acetate toward baseline was more rapid with this decreased dose (Fig. 4B). Plasma acetate levels were significantly elevated above baseline in both males and females at both 5 and 15 min post‐injection. By 30 min post‐injection, only the males had plasma levels that were elevated above baseline. Notably, at 15 min post‐injection, there was a significant difference between the plasma acetate levels in male and female mice.

**Figure 5:**
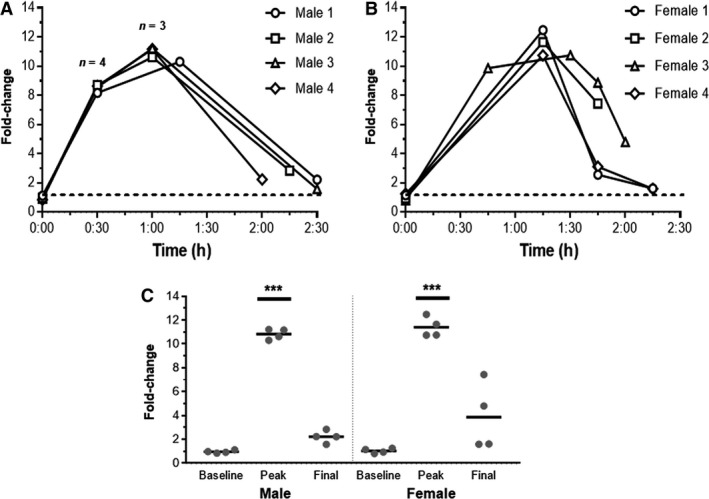
Urine acetate elevates following acetate IP injection. Sodium acetate was delivered at 1 g/kg, IP to male (A) and female (B) mice (*n *=* *4 and 4) following a baseline collection. Urine was collected, when available, at 15 min intervals after injection. Each mouse is individually plotted. In all cases, the first urine sample collected post‐injection showed an increase in acetate, indicating renal clearance of acetate from plasma. The collective fold changes in males and females demonstrate a statistically significant increase (one‐way ANOVA) in acetate at the “peak” timepoint (C). ***P <* *0.001

Finally, we focused on the dose and route which caused the greatest transient increase in plasma acetate (1 g/kg IP) to investigate two additional areas; first, we measured urinary acetate following the 1 g/kg IP dose of sodium acetate to determine if acetate may be cleared from systemic circulation, in part, by the kidneys. All mice reached peak acetate concentration in their urine between 1 and 1.5 h post‐injection (Fig. 5). It should be noted that mice were not catheterized, but rather urine samples (when available) were collected at 15 min intervals. In all cases, the first urine sample collected post‐injection showed an elevation in acetate. By 2.5 h, most of the mice had urinary acetate values near baseline. The average male peak was ~10.8‐fold higher than baseline and the female peak was ~11.4‐fold higher than baseline; both were significantly elevated above their respective baselines (Fig. 5B).

**Figure 6:**
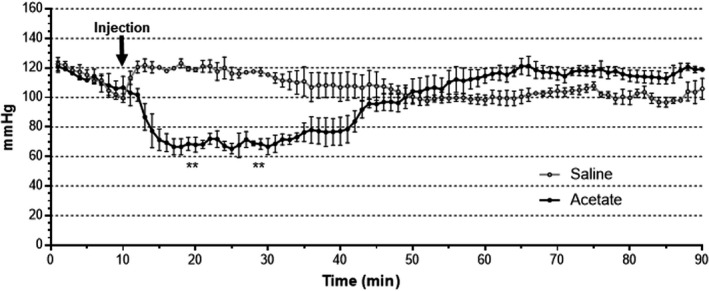
MAP decreases following acetate IP injection. Sodium acetate, or a volume‐matched saline control, was delivered at 1 g/kg IP to male mice following a baseline pressure reading. MAP was significantly lower in the acetate group at minutes 19, 21, 28, and 29 (*n *=* *3, *t*‐test, saline vs. acetate, *P *<* *0.05 using Bonferroni correction for multiple comparisons). MAP, mean arterial pressure.

We have previously reported that intravenous SCFA delivery causes hypotension in mice (Pluznick et al. 2013). Thus, we also used the 1 g/kg IP dose of acetate to determine if the timecourse of changes in plasma acetate mirror changes in a physiological parameter. For this experiment, we delivered sodium acetate 1 g/kg by IP injection in three male mice with implanted telemetry devices for blood pressure measurement. At 12 weeks of age, we administered (on separate days) a saline IP injection or a 1 g/kg sodium acetate injection (Fig. 6). MAP was not different between the two groups (*t*‐test, saline vs. acetate) before injection (minutes 1–10), but was significantly lower in the acetate group at minutes 19, 21, 28, and 29 (*t*‐test using Bonferroni correction for multiple comparisons). Conversely, saline caused only a transient increase in MAP, presumably as a stress response following injection.

## Discussion

Exogenous administration of SCFAs has been widely used to implicate SCFA signaling pathways in physiological processes. The administration of acetate has been shown to modulate appetite suppression, blood pressure regulation, and changes in immune function in vivo (Samuel et al. 2008; Maslowski et al. 2009; den Besten et al. 2013; Pluznick et al. 2013; Trompette et al. 2014). While these physiological responses are well characterized, typically it has not been possible to correlate these modulations with changes in plasma acetate levels. Here, we use a colorimetric kit for acetate detection to perform timecourse evaluations of relative changes in systemic acetate. We validated this kit for detection of acetate in biological samples by demonstrating its relative specificity for acetate at physiological and supraphysiological concentrations. However, it should be noted that this kit has clear limitations. The advantages of EI‐GC MS over the microplate assay include quantitative measurements (*μ*mol/L quantities rather than relative amounts) and the capability of EI‐GC MS to measure multiple SCFAs, not only acetate. Thus, the colorimetric acetate kit cannot function as a replacement for EI‐GC MS; however, due to the small volume of sample required (1 *μ*L plasma vs. 100 *μ*L), it can enable the answering of specific questions, especially when it is necessary to perform a timecourse measurement. In this study, we utilized this kit to delineate for the first time the timecourse of change in systemic acetate after delivery in DW, by OG, and by IP injection.

We were surprised to find that delivering acetate via DW did not cause a detectable change in plasma acetate levels (Fig. 2A–B), even when we withheld water to encourage mice to drink a larger quantity at a specific time (Fig. 2C). Nevertheless, administration of acetate in DW at this level, and at lower levels, has been correlated with physiological effects (Piekarska et al. 2011; Kim et al. 2013). For example, it was shown that mice treated with 200 mmol/L acetate in the DW following *C rodentium* infection had increased extracellular signal‐regulated kinase phosphorylation in the colon, and that this increase in phosphorylation was dependent on the G‐protein–coupled receptors Gpr41 and Gpr43 (Kim et al. 2013). Similarly, administration of DW acetate at 200 mmol/L was protective against colitis in wild‐type mice but not in Gpr43^−/−^ mice (Maslowski et al. 2009). Of note, in a paper published as we were completing our study, Lucas et al. (2018) performed an extended 8‐week treatment with 150 mmol/L sodium acetate, and other SCFAs supplemented in DW and found that these improved bone formation in mice. Following their treatment regimen, they did quantify acetate in mouse serum and found no significant increase in acetate concentration, although they did find an increase in total SCFAs.

There are at least two potential explanations for why our group and others see no changes in plasma acetate, yet clear physiological effects have been seen with similar exogenous doses. First, it is possible that there are small, transient changes in plasma acetate which may be sufficient to induce a response. In this scenario, it is possible that the timepoints we chose are “missing” these transient increases. Another possibility is that the effects seen when acetate is administered though DW is mediated by sensors which reside in the gut or by the enteric nervous system. In this schema, an effect of acetate DW implies that the initial site of action for acetate is the gut itself, and therefore, that elevating colonic acetate is sufficient to see an effect. This possibility fits well with several of the above examples, where the phenotypes observed were also associated with the colon.

In contrast to our results with DW delivery, we saw a clear dose‐dependent and sex‐dependent increase in plasma acetate with both OG delivery and IP injection. It is worth noting that the increase in plasma acetate with OG delivery implies that the “problem” with DW delivery is not the route of administration, but rather the dose. Both the OG doses and IP injections generally elicited an increase that was approximately threefold the baseline measurement of acetate, although the 1 g/kg IP injection exceeded this threefold mark in males. However, it is interesting to note that the dose‐dependent increases seen are not linear with the increase in dose; that is, the 1 g/kg dose does not elicit plasma levels that are double that of the 0.5 g/kg dose. This implies that other factors—that is, absorption kinetics and/or the rate of clearance from the plasma—limit the ability to further increase plasma levels. In addition, the length of time that acetate remains elevated above baseline varies with both dose and delivery method.

It is currently unknown how acetate is cleared from the plasma. Here, we report that acetate accumulates in the urine quickly after IP injection, implying that renal excretion is a primary route by which acetate is cleared from the systemic circulation (Fig. 5). Acetate is small enough to be freely filtered; assuming that it is not bound to plasma proteins, it may be cleared by renal filtration.

Finally, to confirm that changes in plasma levels of acetate correlate with changes in physiological parameters, we also measured blood pressure after acetate was delivered by IP injection (Fig. 6). The timing of the drop in blood pressure correlates with the changes in plasma acetate; the return of blood pressure toward baseline before plasma acetate levels recover likely reflects the large number of blood pressure regulatory mechanisms working to restore blood pressure (Guyton et al. 1972).

In sum, we report here for the first time a clear timecourse for changes in plasma acetate after exogenous, in vivo delivery with doses and methods commonly used in the field (Egorin et al. 1999; Sakakibara et al. 2006; Yamashita et al. 2007; Ge et al. 2008; Kimura et al. 2011; Carvalho et al. 2012; Korecka and Arulampalam 2012; Lin et al. 2012; Frost et al. 2014; Ciarlo et al. 2016; Park et al. 2016; Jia et al. 2017; Mizuno et al. 2017; Li et al. 2018). We find that circulating acetate levels are not measurably increased when acetate is delivered via DW, but there are transient increases in plasma acetate after administration by OG or IP injection. Delivery of exogenous acetate to mice is a common experimental protocol, and this work provides useful data to better understand how these manipulations are altering systemic acetate, and over what timecourse.

## Conflicts of Interest

No conflicts of interest, financial or otherwise, are declared by the authors.
